# Disentangling the effects of working memory, language, parental education, and non-verbal intelligence on children’s mathematical abilities

**DOI:** 10.3389/fpsyg.2014.00415

**Published:** 2014-05-09

**Authors:** Violeta Pina, Luis J. Fuentes, Alejandro Castillo, Sofia Diamantopoulou

**Affiliations:** Departamento de Psicología Básica y Metodología, Facultad de Psicología, Regional Campus of International Excellence “Campus Mare Nostrum,” Universidad de MurciaMurcia, Spain

**Keywords:** mathematics, working memory, language, scholar children, non-verbal intelligence

## Abstract

It is assumed that children’s performance in mathematical abilities is influenced by several factors such as working memory (WM), verbal ability, intelligence, and socioeconomic status. The present study explored the contribution of those factors to mathematical performance taking a componential view of both WM and mathematics. We explored the existing relationship between different WM components (verbal and spatial) with tasks that make differential recruitment of the central executive, and simple and complex mathematical skills in a sample of 102 children in grades 4–6. The main findings point to a relationship between the verbal WM component and complex word arithmetic problems, whereas language and non-verbal intelligence were associated with knowledge of quantitative concepts and arithmetic ability. The spatial WM component was associated with the subtest *Series*, whereas the verbal component was with the subtest *Concepts*. The results also suggest a positive relationship between parental educational level and children’s performance on *Quantitative Concepts*. These findings suggest that specific cognitive skills might be trained in order to improve different aspects of mathematical ability.

## INTRODUCTION

Mathematics is one of the essential tools of everyday life. A clear relationship has been established between poor mathematical performance and lower occupational status, higher unemployment rates, and reduced chance of promotion (Geary, 2011). The acquisition of mathematical abilities is not only one of the foundation stones of the schooling process, but it also affects society in general (Gross et al., 2009). Therefore, understanding the factors that have a decisive influence on children’s performance in mathematics, as well as efficient ways of amelioration of such deficits is of major relevance from an educational, clinical, and social approach.

## A COMPONENTIAL APPROACH TO WORKING MEMORY AND MATHEMATICAL ABILITY

One way of undertaking such aim is to investigate the cognitive skills that better predict children’s performance in mathematical tests, mathematical attainment at school or both. Many studies claim that mathematical abilities are mainly related to working memory (WM; e.g., Geary, 1993; Alloway et al., 2005; Passolunghi and Pazzaglia, 2005; Passolunghi et al., 2007; Passolunghi and Cornoldi, 2008; Holmes et al., 2009; Alloway and Alloway, 2010; Swanson, 2011), and training in WM skills seems to have beneficial effects on some mathematical abilities (see Klingberg, 2010 for a review). However, both WM and mathematical ability are not unitary entities.

Working memory has several components with different functional roles. For instance, in the WM model of Baddeley (1986, 2000) a central executive system would control for how information from different stores is manipulated and integrated according to task demands. Regarding the stores, the phonological loop holds material in a phonological code, and the visual–spatial sketchpad, or according to Logie (1995) the visual–spatial WM, both holds and manipulates visual and spatial information. Regarding this latter component of WM, there is evidence from neuropsychology that patients might show a selective deficit in the visual component (e.g., Carlesimo et al., 2001) or in the spatial component (Hanley et al., 1991; Luzzatti et al., 1998). Interestingly, it seems that it is the spatial rather than the visual component that better predicts mathematical performance (see Mix and Cheng, 2012, for a review). For instance, Passolunghi and Mammarella (2010) showed that children with low scores in problem-solving tasks showed poor performance in the spatial WM tasks but not in the visual ones. However, in a similar study with children with mathematics learning disabilities, the authors showed that these children performed poorly only in the spatial tasks with high attentional demands (Passolunghi and Mammarella, 2012), that is, in tasks that tap the executive control system more than in tasks that only require passive recall of information. The above results fit better with the idea of WM as a continuum of components that require to both modify and integrate information (Cornoldi and Vecchi, 2003).

Mathematical ability should also be considered as a componential entity. Main evidence comes from neuropsychological (Dehaene et al., 1999; Delazer, 2003; Castelli et al., 2006; Demeyere et al., 2012) and functional brain imaging (Zamarian et al., 2009) studies, showing that different mathematical deficits and brain networks are involved in different components of numeric processing. Of special relevance to the mathematics componential issue are intervention studies in educational settings (see Cohen Kadosh et al., 2013 for a review). For instance, Dowker and her colleagues (Dowker, 2001; Dowker and Sigley, 2010; Holmes and Dowker, 2013) have recently developed targeted interventions for children with difficulties in mathematics. Part of their intervention success might come from the fact that mathematics is seen as involving independent and separately developing skills and processes. Children are individually assessed on the different components and the intervention is tailored according to the child’s performance in those components. What these studies reveal is that the mathematics abilities are not organized hierarchically, and some children might perform well at supposedly more difficult tasks and worse at supposedly easier tasks (Dowker, 2005). Nonetheless, although independently developed, some components may form the basis for better learning other specific components, something that is reflected on how the multiple mathematics abilities are taught and learned. For instance, Andersson (2008) points out that basic mathematical skills such as counting are the indispensable basis for a later acquisition of basic arithmetic tasks, and more complex tasks such as multi-digit calculation and word problem solving would be facilitated by the mastery of basic mathematic skills and the conceptual understanding of the principles of calculus. The choice of the mathematics tasks for the present study was done on the basis of the above principles.

## DIFFERENT COMPONENTS OF WM AND MATHEMATICAL ABILITY

On the basis of the aforementioned studies, it is clear that an appropriate approach to understanding what factors contribute to performance in mathematics, should examine the specific effects of both verbal and spatial WM on children’s mathematical ability (for a review see Berch, 2008). Regarding the verbal component of WM, there is not conclusive evidence for a relationship with mathematics performance. For instance, McLean and Hitch (1999) showed that children with poor arithmetic abilities had impaired spatial WM and aspects of executive processing, while their verbal WM proved to be unaffected. Some studies have shown that the phonological dimension of language is not involved in mathematical performance (Passolunghi et al., 2007), while other studies suggest an important relationship between them (Koponen et al., 2007; Simmons et al., 2008). For instance, De Smedt et al. (2010) in a sample of children 9- to 11-years old showed that phonological awareness is related to mathematical problems that require retrieval from long-term memory such as small additions, subtractions, and multiplications, but it is not related to mathematical problems that require procedural strategies such as large additions and subtractions. However, that study did not control for WM function.

Regarding the relation between the spatial component of WM and mathematical performance findings are inconclusive. On the one hand, several studies have shown a clear relation between spatial WM and different mathematics abilities in an ample range of participants, including typically developing children, special populations, high school students, and adults (for a review, see Mix and Cheng, 2012). On the other hand, null results have also been reported. Bull et al. (1999) did not find differences in spatial WM between 7-year-old children scoring high and low in mathematical ability. Alloway and Passolunghi (2011) evaluated the performance of 7- to 8-year-old children in tasks such as quantitative discrimination, numeric ranking, numeric production, number operation, and arithmetic and their findings suggest that spatial WM does not make any contribution to these mathematical tasks. Finally, in a longitudinal study, Swanson (2011) examined which grade 1 cognitive abilities make unique contributions to problem-solving accuracy in grade 3 and found that, with control for other cognitive abilities, only the central executive and verbal WM significantly predicted grade 3 word problem-solving accuracy.

In a review article, Geary (2011) established that the relevance of the verbal and the spatial components of WM vary depending on the content of the mathematical ability that is being evaluated. Processes such as counting and solving mathematical word problems, which involve the articulation of numbers, are supported by the verbal component of WM. By contrast, processes involved in the number line and translating word problems into mathematical equations seem to be supported by the spatial component. Furthermore, it seems that both the executive and spatial aspects of WM are relevant in the acquisition and application of new concepts and skills, whereas the verbal component is more relevant once the skill has been acquired (Raghubar et al., 2010).

All the above results highlight the relevance of examining the relationship between different aspects of WM and different areas of mathematics rather than general mathematical ability or a particular mathematical ability.

## CONTRIBUTION OF OTHER FACTORS TO MATHEMATICAL ABILITIES

Recent studies suggest that the role of WM might be captured by other academic or cognitive factors. For instance, Lee et al. (2004) in a study with children in the fifth grade, found that those with a higher IQ and better reading and vocabulary abilities showed better mathematical performance when solving problems (see also Grimm, 2008). WM did not explain much variance in problem-solving accuracy when such factors were entered into the regression analyses (but see Swanson, 2011). Krajewski and Schneider (2009) assessed a sample of children from their last year in kindergarten until the beginning of grade 3. Their results suggested that the relationship between spatial WM and mathematical performance is not direct; rather, spatial WM influences school achievement through higher-order domain-specific precursor variables such as the understanding of the link between quantity and number word. The previously mentioned study by Alloway and Passolunghi (2011) with 7- and 8-year-old children showed that vocabulary predicts performance across mathematical tasks (quantity discrimination, number ranking, number operations, and arithmetic) regardless of age. In spite of the wide variety of studies measuring verbal ability, WM, and mathematics, the Alloway and Passolunghi’s (2011) study was, to our knowledge, the only research that examined the relationship between the different components of WM, different mathematical areas, and verbal ability.

Intelligence seems also to play a role in the contribution of WM to mathematical performance. Although WM seems to be a better predictor of mathematical accuracy, compared to intelligence (see Swanson, 2011), IQ has been found to account for a proportion of unique variance in mathematical performance (e.g., Swanson and Beebe-Frankenberger, 2004; Alloway and Alloway, 2010). However, the relationship between IQ and mathematical performance has not always been supported in the existing literature (Alloway, 2009). Moreover, the relation between WM, IQ, and mathematical ability might be further contaminated by the fact that WM tasks are often included in intelligence tests such as the digit-span task.

As Gifford and Rockliffe (2012) point out, there are many factors that might contribute to children experiencing difficulties in mathematics. So far we have highlighted the relevance of cognitive factors such as WM, but there is no doubt that other cognitive abilities, mainly involving language, may also play an important role in children’s difficulties with mathematics. Finally, environmental factors have also been shown to play a relevant role in children’s performance at school (e.g., Levels et al., 2008). Several studies have found a relation between family socioeconomic status (SES) and academic achievement in general (e.g., Aunio and Niemivirta, 2010), and mathematical performance in particular (e.g., Jordan and Levine, 2009). The more educated families are more likely to create richer intellectual environments increasing children’s opportunities for learning.

## THE PRESENT STUDY

In the present study we aimed to examine the relationship between the verbal and the spatial components of WM and different areas of mathematical abilities in a typically developing sample of children aged 9–13 years. We chose the particular age range mainly because of two reasons. First, because it seems that both spatial and verbal WM are less differentiated in children younger than 8 years (Hale et al., 1997). Second, it is around this age that children start to engage in phonological rehearsal (Gathercole et al., 1994; Gathercole, 1998; Tam et al., 2010; Gaillard et al., 2011). Also, there is an increase in the verbal abilities of children from age 7 that allows them to make a wider use of verbal information (Hitch et al., 1988). This in turn reflects on an increasing ability to solve mathematical problems with long and complex verbal instructions.

An important characteristic of the present research is that we adopted a componential approach to assess the contribution of different components of WM on several areas of mathematics. For the spatial component of WM we used computerized versions of both the forward and backward condition of the Corsi blocks task. For the verbal component of WM we used both the forward and the backward digit-span test (WISC-IV; Wechsler, 2004). We assume that whereas the forward versions of both tasks require both storage and recovery information from WM with minimal demands on the central executive, the backward versions require greater demands of attentional control and therefore a higher involvement of the central executive (Cornoldi and Vecchi, 2003; Passolunghi and Mammarella, 2010). To assess children’s competence in mathematics, we selected three tests that reflect several levels of mathematical abilities. Two tests, *Fluency* and *Quantitative Concepts*, were selected from the Spanish version of Woodcock Johnson III Tests of Achievement (Muñoz-Sandoval et al., 2005), and the third test was *Arithmetic* from the WISC-IV (Wechsler, 2004). On the basis of previous findings we advanced the following hypotheses.

Regarding the verbal component of WM we expected it to be related to the mathematical abilities that rely on memory retrieval facts (e.g., Raghubar et al., 2010; Geary, 2011). It is assumed that exact arithmetic retrieval is a language-based skill (Dehaene et al., 1999), and therefore the verbal component of WM should show unique variance contribution to children’s performance on very simple arithmetic operations. Here we measured such mathematical ability through the *Fluency* test.

Whereas the forward digit span test requires minimal processing load, recalling digits in backward order increases the processing load and makes higher demands on the central executive, and therefore it is a better measure of WM (Isaacs and Vargha-Khadem, 1989; Alloway and Alloway, 2010 see also Vandierendonck et al., 2004). Thus, we expect the verbal component of WM, as indexed by the backward version of the digit span test, to contribute with unique variance to word problem solving, as the central executive has been related to that mathematical ability (Swanson, 2011). Word problem solving was assessed here through the *Arithmetic* test.

Mathematical competence requires knowledge of mathematical vocabulary, symbols, formulas, and transcoding from a symbolic form to a verbal form. Higher abilities to maintain and manipulate such verbal knowledge in WM should relate to better performance in an ample range of mathematical problems. We measured children’s knowledge about mathematical concepts with the *Quantitative Concepts* test, mainly the subtest *Concepts.*

Other mathematical problems require spatial representation of partial operations, as when children are asked to solve numerical sequences. In numeric sequence problems a transformation of the numeric sequence into a mathematical formula is required to solve the task. Those operations might require a higher involvement of the central executive to use and maintain spatial information in WM, and therefore we expect a strong influence of that WM component on such mathematical problems. We measured such children’s ability through the subtest *Series* of the *Quantitative Concepts* test.

With regard to the effects of intelligence on math performance, due to its relation to the central executive, we expect it to contribute with unique variance on those mathematical tasks that make greater demands on the central executive. Given that we used only a non-verbal intelligence test we expect intelligence to share some processing operations with the spatial WM tasks, but not with the verbal WM ones, and therefore to have some influence in the relation between spatial WM and mathematical abilities. Regarding language, on the basis of previous findings (e.g., Alloway and Passolunghi, 2011), we expected language to only significantly predict high-level mathematical problems that require a high level of language comprehension.

## MATERIALS AND METHODS

### PARTICIPANTS AND PROCEDURE

One hundred two typically developing children (46 boys) attending primary school in grades 4–6 (mean age = 10 years, SD = 11 months) took part in the study. This sample was part of a larger sample included in a study that aimed at validating the math tests of the Spanish version of Woodcock Johnson III Tests of Achievement (Muñoz-Sandoval et al., 2005), for its use in Spain (see **Table 1** for a detailed description of the sample by gender and school grade). The original sample consisted of 424 children (216 boys) who attended primary school and were in grades 1–6 (mean age = 9 years, SD = 2 months) from eight different schools in the region of Murcia, in southeastern Spain (for a detailed description of the original sample, see Diamantopoulou et al., 2012). The original sample was assessed with a battery of intelligence and math tests in spring semester (Time 1). Children attending grades 4–6 in the same schools were assessed with a battery of cognitive and math tests in the fall semester (Time 2). This study is based on data from Time 1 and Time 2. Written informed consent was obtained from the parents, and the participants gave oral consent before the testing sessions. Informed consent documentation was sent to 205 parents whose children were in grades 4, 5, and 6 and who had completed Time 1 previously. We obtained 157 responses after two reminders. Of those, we collected 102 questionnaires from parents; thus, the data analyses were conducted on those children’s scores from which we had the complete testing.

**Table 1:** Descriptive data.

Grade	*n* (boys)		Age (months)	
		*M*	SD	Range
4	36 (16)	113.1	3.7	106–123
5	27 (12)	125	5.1	118–139
6	39 (18)	136.7	4.5	130–150
Total	102 (46)	125.3	11.1	106–150

We obtained approval from the bioethics committee of the University of Murcia (Spain). Trained assistants administered tests individually in a counterbalanced sequence to avoid systematic variations arising from the order of administration. We used random ordering protocols with a table of random digits for each child to achieve counterbalance.

### MEASURES

#### Mathematic abilities

At Time 2, we assessed children’s math ability with the math Fluency and Quantitative Concepts tests of the Spanish version of the WJ-III ACH battery, which was primarily validated for its use with participants from 6 to 13 years in Spain (see Diamantopoulou et al., 2012), and with the Arithmetic test of the Spanish version of the WISC-IV (WISC-IV; Wechsler, 2004). For all tests we used summed scores to assess performance with ascending numbers indicating better performance.

***Fluency.*** This test measures the ability to solve simple one-digit operations such as addition, subtraction, and multiplication facts. The test consists of a series of 160 simple arithmetic problems in a participant response booklet and the child is asked to complete as many as possible in a 3-min time limit. The dependent variable was the total correct responses obtained within the time limit.

***Quantitative concepts.*** This test measures knowledge of mathematical concepts, symbols, and vocabulary. It consists of two subtests, *Concepts* and *Series*. In the subtest *Concepts*, participants are asked to count or identify numbers, shapes (e.g., the multiplication and division symbols), and sequences (e.g., what number comes after 37?), and to know mathematical formulas and terms (e.g., round a number to the closest hundred). It consists of 34 items of increasing difficulty, which are read to the child. In the subtest *Series*, participants are asked to look at a series of numbers, figure out the pattern and provide the missing number in the series (e.g., the series 4 7 10 _). This test consists of 23 problems of ascending difficulty. The dependent variables of the two subtests were the summed scores of correct responses with ascending numbers indicating better performance. Internal consistency of the measure *Quantitative Concepts* in the large original sample was excellent, with *α* = 0.92 from 6 to 13 years.

***Arithmetic.*** We also assessed arithmetic ability at Time 2 with the arithmetic ability subtest of the Spanish version of the Weschler intelligence test for children (WISC-IV; Wechsler, 2004). For this test, the child was asked to solve a maximum of 34 arithmetic problems of ascending difficulty, presented orally within a limited time. Children were asked to infer the mathematical operation that each problem requires (e.g., addition, subtraction, multiplication, or division), or more complex operations such as rules of third. We used the summed scores of the test as the dependent variable. Ascending scores indicate better performance. The Spanish version of this test has an excellent internal consistency, with alphas ranging from *α* = 0.88 to *α* = 0.81 for ages 9–13 years.

#### Non-verbal intelligence

We assessed non-verbal Intelligence with the Spanish version of the Matrices subtest of the Kaufman brief intelligence test (Kaufman and Kaufman, 1990) in Time 1. In this subtest children are presented a series of abstract and figurative visual stimuli and they need to select the appropriated response from a multiple-choice selection. This subtest has been related to fluid intelligence. Internal consistency for the Spanish version of this test ranges from *α* = 0.87 to *α* = 0.83, for ages 9–13 years (Kaufman and Kaufman, 1990). We used the summed scores of the test as a measure of non-verbal intelligence. Ascending scores indicate better performance.

#### Working memory

At Time 2 we assessed spatial WM with a computerized version of the Corsi blocks task (Kessels et al., 2000, 2008), and verbal WM with the digit span task of the WISC-IV (Wechsler, 2004).

***Spatial WM.*** In the computerized version of the Corsi blocks task we used to test spatial WM, stimuli were presented on a 15″ color monitor of an IBM laptop with Windows XP Professional at 1024 × 768 pixels resolution. The distance of the participant to the screen was 60 cm. A set of nine identical 30 × 30 mm white squares were presented irregularly positioned on a black background with a white frame. The distance between squares on the screen was 1.28 relative standard positions. All participants completed four practice trials before the testing trials. Practice trials provided a smiley face as feedback if the answer was right and a sad face if it was wrong, shown for 300 ms; this feedback was not included in testing trials. Next, the testing session started. In a standard testing trial, the nine white squares appeared on the black background with a white frame. After 1 s, the squares were individually highlighted in sequence by changing the color to red for 1 s, without any inter-square time. When the sequence finished, immediately the nine white squares appeared on the black background with a red frame. At this point, the participants were asked to reproduce the sequence of the highlighted squares in the order in which they had seen them (forward) or in the reverse order (backward) by clicking on the squares with the computer mouse. Each time a square was clicked, it turned red for 200 ms to confirm that the participant had clicked. After an inter-trial interval of 1 s, the next trial started. Testing stopped when the participants made two consecutive mistakes.

All the participants completed first the forward and then the backward condition. The sequences of forward trials are reported in Kessels et al. (2000), and the sequences of backward trials are derived from Kessels et al. (2008). The length of the number of highlighted squares that children were asked to remember for each trial was between 2 and 9 in the forward condition and between 2 and 8 in the backward condition. There were two trials for each length (two trials with two highlighted blocks, two trials with three highlighted blocks, and so on). The test consisted of a maximum of 18 experimental trials in the forward condition and a maximum of 16 experimental trials in the backward condition. We used the summed scores of the test as a measure of children’s spatial WM. Ascending scores indicates better performance.

***Verbal WM.*** We administered both the forward and the backward digit-span tests of the Spanish version of the Wechsler intelligence test for children (WISC-IV; Wechsler, 2004) at Time 2. The participants were asked to recall a sequence of spoken digits in either the same (forward condition) or the reverse (backward condition) order. All the participants completed first the forward and then the backward condition. The internal consistency of the Spanish version of these tests proved to be excellent, with alphas ranging from *α* = 0.73 to *α* = 0.77 (forward test) and *α* = 0.77 to *α* = 0.78 (backward test), for 9- to 13-year-old children. A maximum of 12 points could be obtained in both the forward and the backward conditions. We used the summed scores of each condition as a measure of children’s verbal WM. Ascending scores indicates better performance.

#### Language

We assessed language ability with the Spanish version of the riddles subtest of the Kaufman assessment battery for children (Kaufman and Kaufman, 1990) at Time 2. This subtest evaluates the ability of participants to infer the name of abstract or concrete verbal concepts from several given characteristics. It consists of 32 items of ascending difficulty that are read to the child. The internal consistency of the Spanish version of this test was excellent with values of *α* = 0.84 to *α* = 0.86 for 9- to 12.5-year-old children. We used the summed scores of the test as a measure of children’s language ability. Ascending scores indicate better performance.

#### Socioeconomic status

Parents completed a questionnaire pertaining to demographic characteristics at Time 1. Parents were asked about family income, educational level, and employment status within a larger questionnaire. Given the drastic economic changes in Spain that occurred during the collection of data, family income, or employment status were likely to bias the true socioeconomic level of the family, so we discarded this variable and focused only on the educational level of the parents. Parents chose among several answers given in the questionnaire sheet, ranging from (1) primary education to university/college degree (9). We classified the answers depending on the level of education.

### STATISTICAL ANALYSES

In the preliminary analyses, we first assessed potential selective attrition effects by conducting a one-way ANOVA comparing the children that completed all testing with those who did not or who did not participate at Time 2 in parental education, non-verbal intelligence, gender (coded as 1 = boys, 2 = girls), and school grade. We then assessed the effects of both gender and grade on all dependent variables by use of two-way ANOVAs. We computed Pearson correlation coefficients between the independent and dependent variables to examine their relations.

In the main analyses, hierarchical regression analyses were conducted to examine the independent effects of non-verbal intelligence, WM, and language on mathematical abilities. All regression analyses included school grade, gender, and parental education as control variables. All predictors were centered prior to analyses to avoid issues of multicollinearity (Aiken and West, 1991). The dependent variables were children’ scores in the tests fluency, quantitative concepts (concepts and series) and arithmetic.

## RESULTS

### PRELIMINARY ANALYSES

Results of the one-way ANOVA we performed to assess potential selective attrition revealed significant effects of parental education [*F*_(2,163)_ = 5.88, *p* = 0.003] and non-verbal intelligence [*F*_(2,200)_ = 4.93, *p* = 0.008]. *Post hoc* analyses (Tukey’s) revealed that the parents of children who had completed all testing had a lower educational level than parents of children who did not participate in Time 2 (*p* = 0.008). The effect size for this difference was medium (*d* = 0.59). Also, we found that children who had completed all the tasks had a lower non-verbal intelligence than children who did not participate in Time 2 (*p* = 0.008). The effect size of this difference was medium (*d* = 0.53). No significant effects were found for grade or gender.

Mean values for all measures organized by school grade and gender are depicted in **Table 2**. We performed two-way ANOVAs with grade (fourth, fifth, and sixth), and gender (boys and girls) as between-participants factors, for each key variable. The results showed significant main effects of grade on the mathematic tests *Fluency* [*F*_(2,96)_ = 8.18, *p* < 0.001], *Quantitative Concepts* [*F*_(2,96)_ = 6.65, *p* = 0.002], *Concepts* [*F*_(2,96)_ = 8.69, *p* < 0.001], and *Arithmetic* ability [*F*_(2,96)_ = 6.07, *p* = 0.003]. We also obtained significant main effects for non-verbal intelligence [*F*_(2,96)_ = 3.85, *p* = 0.02], the forward condition of the Corsi blocks task [*F*_(2,96)_ = 3.6, *p* = 0.031], the backward condition of the Corsi blocks task [*F*_(2,96)_ = 4.77, *p* = 0.011], the forward condition of the digit-span task ([*F*_(2,96)_ = 7.66, *p* < 0.001], and language [*F*_(2,96)_ = 12.09, *p* < 0.001]. As shown in **Table 2**, all the above abilities increased with the school grade. *Post hoc* analyses (Tukey’s) revealed that fourth graders performed worse than fifth graders in the tests Fluency and Concepts, and also in the forward condition of the digit-span task (*p* < 0.05). We also found that fourth graders performed worse than sixth graders in all tasks, except for the backward condition of the digit-span task. Regarding gender effects, we found that girls performed better than boys in the backward condition of the digit-span task [*F*_(1,96)_ = 5.07, *p* = 0.03]. The effect of these gender differences was medium (*d* = 0.48). There were also significant interactions between grade and gender for the test *Series* [*F*_(2,96)_ = 3.78, *p* = 0.026], indicating that boys outperformed girls just in grade 5 and for the backward condition of the Corsi blocks task [*F*_(2,96)_ = 9.85, *p* < 0.001], indicating that boys performed worse than girls just in grade 4. Accordingly, we included gender and grade as control variables in the regression analyses (see Results).

**Table 2:** Mean total scores by grade and gender for all measures.

Grade		Parental education	Non-verbal IQ	Corsi task forward	Corsi task backward	Digit span forward	Digit span backward	Language
		*M* (SD)	*M* (SD)	*M* (SD)	*M* (SD)	*M* (SD)	*M* (SD)	*M* (SD)
4	All	4.75 (2.71)	24.94 (4.78)	7.03 (1.23)	6.86 (2.19)	6.86 (1.12)	5.97 (1.23)	17.81 (2.36)
	Boys	4.09 (2.27)	23.5 (4.55)	6.5 (1.26)	5.31 (1.78)	6.88 (1.09)	5.5 (0.96)	16.88 (2.5)
	Girls	5.27 (2.97)	26.1 (4.76)	7.45 (1.05)	8.1 (1.65)	6.85 (1.18)	6.35 (1.31)	18.55 (2.01)
5	All	4.35 (2.47)	26.59 (4.74)	7.59 (1.45)	6.96 (2.12)	7.96 (1.67)	6.52 (0.97)	19.67 (3.32)
	Boys	4.79 (2.33)	26.42 (4.98)	7.92 (1.5)	7.42 (1.88)	7.42 (1.62)	6.33 (0.98)	20.75 (3.47)
	Girls	4 (2.6)	26.73 (4.71)	7.33 (1.4)	6.60 (2.29)	8.4 (1.64)	6.67 (0.98)	18.80 (3.03)
6	All	3.88 (2.52)	27.97 (4.98)	7.77 (1.4)	7.95 (1.7)	8.05 (1.39)	6.51 (1.83)	21.26 (3.68)
	Boys	4.19 (2.61)	27 (5.03)	7.83 (1.2)	8.17 (2)	7.89 (1.6)	6.11 (1.23)	21.61 (3.77)
	Girls	3.62 (2.47)	28.81 (4.9)	7.71 (1.59)	7.76 (1.41)	8.19 (1.21)	6.86 (2.2)	20.95 (3.65)

**Grade**		**Fluency**	**Quantitative concepts**	**Series**	**Concepts**	**Arithmetic**		
		***M* (SD)**	***M* (SD)**	***M* (SD)**	***M* (SD)**	***M* (SD)**		

4	All	58.57 (15.86)	33.61 (3.16)	13.5 (1.83)	20.05 (2.08)	19.08 (2.85)		
	Boys	59.94 (19.74)	32.88 (3.72)	12.94 (2.29)	19.94 (2.01)	19 (2.99)		
	Girls	57.45 (12.36)	34.2 (2.59)	13.95 (1.23)	20.15 (2.18)	19.15 (2.82)		
5	All	70.41 (15.65)	35.7 (4.06)	14.07 (1.49)	21.63 (3.09)	20.52 (3.04)		
	Boys	76.08 (18.44)	37.08 (5.02)	14.92 (1.5)	22.17 (3.99)	22 (2.73)		
	Girls	65.87 (11.75)	34.6 (2.82)	13.4 (1.12)	21.2 (2.18)	19.33 (2.82)		
6	All	75.05 (21.11)	36.41 (3.64)	14.18 (2.33)	22.23 (1.81)	21.46 (3.23)		
	Boys	77.17 (17.63)	36.89 (4.51)	14.61 (2.77)	22.28 (2.24)	21.83 (2.57)		
	Girls	73.24 (23.98)	36 (2.74)	13.81 (1.86)	22.19 (1.4)	21.14 (3.74)		

Bivariate correlations between children’s scores in all measures are provided in **Table 3**. The strength of the associations between non-verbal intelligence, forward condition of the Corsi blocks task, backward condition of the Corsi blocks task, forward condition of the digit-span test, backward condition of the digit-span test, and language (in italic) was low to medium, with significant correlation coefficients ranging from *r* = 0.19 to *r* = 0.48, *p* < 0.05, with the exceptions of the association between the forward condition of the digit-span test and non-verbal intelligence (*r* = 0.14, *ns*); the forward condition of the digit-span test and the backward condition of the Corsi blocks task (*r* = 0.16, *ns*); the backward condition of the digit-span test and the backward condition of the Corsi blocks task (*r* = 0.15, *ns*); and finally, between language and the backward condition of the digit-span test (*r* = 0.14, *ns*). For the mathematics measures, the significant correlations ranged from *r* = 0.37 to *r* = 0.88, *p* < 0.01. The strength of the associations between the mathematics measures and non-verbal intelligence, the forward condition of the Corsi blocks task, the backward condition of the Corsi blocks task, the forward condition of the digit-span test, the backward condition of the digit-span test, and language (in bold) went also from low to medium, with significant correlation coefficients ranging from *r* = 0.20 to *r* = 0.51, *p* < 0.05. Parental education correlated significantly with the backward condition of the Corsi blocks task and language (*r* = 0.28 and *r* = 0.31, *p* < 0.01, respectively).

**Table 3:** Correlations among all measures.

	1	2	3	4	5	6	7	8	9	10	11	12
1. Parental education	1											
2. Non-verbal IQ	0.13	1										
3. Corsi task forward	0.14	27^**^	1									
4. Corsi task backward	0.28^**^	0.40^**^	0.41^**^	1								
5. Digits span forward	0.03	*0.14*	0.19^*^	*0.16*	1							
6. Digits span backward	-0.01	27^**^	0.26^**^	*0.15*	0.35^**^	1						
7. Language	0.31^**^	0.48^**^	39^**^	0.47^**^	0.22^*^	*0.14*	1					
8. Fluency	0.02	0.23^*^	0.27^**^	0.20^*^	0.33^**^	0.26^**^	0.36^**^	1				
9. Quantitative concepts	0.17	0.50^**^	0.27^**^	0.41^**^	0.32^**^	0.29^**^	0.51^**^	0.46^**^	1			
10. Series	0.09	0.36^**^	0.21^*^	0.32^**^	0.21^*^	0.22^*^	0.36^**^	37^**^	0.81^**^	1		
11. Concepts	0.19	0.47^**^	0.25^*^	0.37^**^	0.31^**^	0.28^**^	0.49^**^	0.41^**^	0.88^**^	44^**^	1	
12. Arithmetic	0.01	0.47^**^	0.32^**^	0.32^**^	0.35^**^	0.40^**^	0.49^**^	0.62^**^	0.57^**^	0.45^**^	0.51^**^	1

* *p < 0.05; **p < 0.01.

### MAIN ANALYSES

**Table 4** depicts the results of the hierarchical regression analyses. As seen in **Table 4**, the three predictors included in the first step accounted for 13% of the explained variance in the test Fluency, but only grade made a significant contribution. The second step accounted for 9% of the explained variance. None of the variables contributed with unique variance to performance in the fluency test.

**Table 4:** Results of hierarchical regression analyses.

	Fluency		Quantitative concepts		Series		Concepts		Arithmetic	
	ΔR2	β	ΔR2	β	ΔR2	β	ΔR2	β	ΔR2	β
School grade		38^**^		0.35^**^		0.16		0.41^**^		0.33^**^
Gender		-0.13		-0.07		-0.09		-0.04		-0.14
Parental education		0.08		0.22^*^		0.11		0.25^**^		0.06
Step	0.13^**^		0.13^**^		0.01		0.18^**^		0.10^**^	
Non-verbal IQ		0.05		29^**^		0.21		0.28^**^		0.26^**^
Corsi task forward		0.08		-0.03		-0.01		-0.04		0.04
Corsi task backward		0.02		0.17		0.18		0.11		0.09
Digits span forward		0.18		0.16		0.13		0.14		0.19^*^
Digits span backward		0.16		0.15		0.13		0.13		27^**^
Language		0.17		0.21		0.19		0.16		0.28^**^
Step		0.09^*^		0.26^**^		0.17^**^		0.17^**^		0.36^**^

* *p* < 0.05; ** *p* < 0.01.

School grade, gender, and parental education made a significant contribution to the explained variance of *Quantitative Concepts*. However, when the two subtests that constitute *Quantitative Concepts* were examined separately, school grade significantly predicted performance in both subtests, whereas parental education only predicted the subtest *Concepts*. The second step of the analysis contributed to another 26% of the explained variance in the test *Quantitative Concepts*. For the two subtests, *Concepts* and *Series,* the second step contributed to 17% of explained variance, but none of the variables significantly predicted performance in these tests.

In the case of *Arithmetic* ability, the first step of the analysis explained 10% of the variance, but only school grade made a significant contribution. The second step accounted for 36% of the explained variance, with non-verbal intelligence, digit-span forward, and backward condition and language making significant unique contributions.

Because some authors have pointed out that intelligence can contribute to the relation of some components of WM and mathematical abilities (e.g., Lee et al., 2004), we repeated the regression analysis removing non-verbal intelligence from the model. The results showed that both the backward condition of the Corsi blocks task and the backward condition of the digit-span task made unique and significant contributions to *Quantitative Concepts* (β = 0.23, and β = 0.20, respectively for the Corsi blocks task and the digit-span task; *p* < 0.05). However, when the two subtests were examined separately, only the backward condition of the Corsi blocks task significantly predicted the subtest *Series* (β = 0.22, *p* < 0.05), and the backward condition of the digit-span test predicted the subtest *Concepts,* although in latter case it was only marginally significant (β = 0.18, *p* = 0.06). The results also showed that language made a significant contribution not only to *Quantitative Concepts* as before (β = 0.32, *p* < 0.01), but also to both *Concepts* and *Series* (both βs = 0.27, *p* < 0.05).

## DISCUSSION

In reviewing the role of WM in mathematics performance, it is apparent that only few studies have looked at the relationships of the different components of WM and different mathematical abilities in a single study. However, on the basis of neuropsychological, neuroimaging and intervention studies, it is clear that different components might be related with some but not all mathematical areas. This componential approach that was taken in the present study constitutes the most important contribution to the existing literature.

As measures of WM we clearly differentiated those that tap the verbal component from those that tap the spatial component. We also employed different WM tasks that have been assumed to make differential recruitment of the central executive (Cornoldi and Vecchi, 2003; Passolunghi and Mammarella, 2012). The forward versions of the Corsi blocks task and the digit-span test were assumed to tap passive coding of spatial and verbal information, respectively, whereas the backward versions would require both holding and manipulating such information in WM, and therefore demand higher level of attentional control. We hypothesized that different components of WM would affect children’s performance on specific rather than general mathematical abilities. Whereas mathematical operations based on retrieval memory facts were expected to be influenced by the verbal component of WM, performance in mathematical word problems was expected to be related with the verbal component of WM that makes greater recruitments of the central executive. The verbal component was also expected to influence performance in mathematical problems that require specific mathematical knowledge. In contrast, the spatial components were expected to affect performance in mathematical problems that require both using and maintaining spatial information in WM, like in numerical sequence problems. Briefly, our results confirmed dissociable effects of both components of WM on mathematical performance. The verbal component measured through the backward condition of the digit-span test related to the mathematical abilities that more relay on verbal competence (*Arithmetic* and *Concepts*). In contrast, the spatial component of WM measured through the backward condition of the Corsi blocks task related to the mathematical operations that require both using and maintaining spatial information to solve the problems (*Series*). We also looked at the contribution of other factors such as non-verbal intelligence, language, and family SES (measured through parental educational level) to mathematical performance, either as factors that explain unique variance or as in the case of non-verbal intelligence as contributing to the relationships between WM and mathematics. As hypothesized, non-verbal intelligence related to the mathematical tests that make greater demands on the central executive (*Arithmetic* and *Quantitative Concepts*), language to the ones that make greater demands on verbal information (*Arithmetic* and *Concepts*), and parental education was related to *Quantitative Concepts*. A more detailed discussion of aforementioned results is presented below.

### WM, INTELLIGENCE, AND MATHEMATICS

A main finding of the present study is that only verbal WM was associated with verbal arithmetic problems (the *Arithmetic* test). That relation was stronger with the digit-span backward condition than with the digit-span forward condition, as it has been shown that more complex arithmetic problems require not only the ability to hold information in memory but also the ability to integrate new information with previously processed one (e.g., holding partial results to integrate them with new data; Swanson, 2011). Contrary to our predictions, digit-span forward condition did not significantly predict *Fluency* scores. Although the observed correlation coefficient between the two variables was larger than the ones showed by the other WM tests (see **Table 3**), this relation vanished in the regression analysis. This lack of relation suggests that mathematical abilities required for successfully performing the *Fluency* test require low language level and might depend more on recovery of number facts from long-term memory (Andersson, 2008).

Regarding the spatial WM component, although the Corsi blocks tasks correlated positively with all mathematical tests, those correlations vanished when these spatial tests entered in the regression analysis together with verbal WM, language, and non-verbal intelligence. This result does not fit with the bulk of evidence that points to a clear relation between spatial abilities and mathematics (Mix and Cheng, 2012), although there are studies that have not found such a unique association (see Swanson, 2011 for a similar finding with word problem solving). There are several possible explanations for a lack of unique contribution of spatial WM to children’s mathematics performance. The spatial component of WM might be involved to a greater extent in the acquisition of basic processes in younger children, but as these processes are acquired, it is the verbal component that takes a more dominant role in the support of arithmetic performance (Raghubar et al., 2010; see present results with the digit-span tests). In line with this argument is the fact that in our applied problems test (*Arithmetic*), the participants were asked to respond to the items with a time limit. Newly acquired knowledge could lead to incorrect responses, as the time needed to implement it would be greater. Correct responses could then arise from already acquired knowledge, thus showing no direct intervention of spatial WM. An alternative account is that the unique contribution of spatial WM to mathematics performance has been captured by other variables such as the non-verbal intelligence test. Note that we used only the “matrices” part of the K-BIT to measure fluid intelligence in our sample, a test that taps spatial reasoning abilities. It should then be expected that the non-verbal intelligence test will affect the relationship between mathematical performance and the backward condition of the Corsi blocks task, as this task is assumed to tap the central executive and requires both holding and integration of spatial information. Accordingly, we observed a stronger correlation between the backward condition of the Corsi blocks task and non-verbal intelligence than between the non-verbal intelligence test and the other WM tasks (see **Table 3**). This account is further supported by the unique contribution of the backward condition of the Corsi blocks task to *Quantitative Concepts* when non-verbal intelligence is removed from the regression analysis (see below for a more detailed explanation).

As hypothesized, non-verbal intelligence showed a prominent role in those mathematical tests that make greater demands on the central executive. The link between non-verbal intelligence and mathematics is more evident as the complexity of the test grows. We found that mathematical areas in continuous learning such as *Quantitative Concepts* and *Arithmetic* are related to non-verbal intelligence. As stated previously, this relationship has not always been supported (e.g., Alloway, 2009). Our findings agree with those reported by Alloway and Alloway (2010), which suggest that, even though there is an association between WM and mathematical achievement, intelligence has a unique contribution to mathematical performance. In addition, our findings support the notion that the relationship between non-verbal intelligence and mathematical performance is determined by the complexity of the task, as simpler tasks such as *Fluency*, which is a result of practice, do not show direct evidence of such a relationship.

The important role of non-verbal intelligence in the present study is further supported by its influence in the relationships between the two components of WM and a particular test of mathematical ability, *Quantitative Concepts*. As mentioned previously, this test is composed of two different subtests, *Concepts* and *Series.* These two subtests share important operational processes that appear to be influenced by the children’s level of fluid intelligence. But they might also require singular processing that is differentially associated with the different components of WM. Thus, *Concepts* requires transcoding from a symbolic and/or visual form to a verbal form, and therefore performance in this test might depend on children’s verbal WM capacity. This is supported by the relation between the backward condition of the digit-span test and the subtest *Concepts*. On the other hand, *Series* requires holding and manipulating a spatial representation of the previous operations, needed to solve the numerical sequence, and therefore performance in this test might depend on children’s spatial WM abilities. This is supported by the relation between the backward condition of the Corsi blocks task and the subtest *Series*.

Briefly, non-verbal intelligence and WM seem to be excellent predictors of performance in those mathematics abilities that require knowledge of mathematical concepts (*Quantitative Concepts*) and solving of word arithmetic problems (*Arithmetic*). However, they do not affect performance of simple calculations that might depend more on recovery of number facts from long-term memory (e.g., Andersson and Lyxell, 2007; Andersson, 2008). This is supported by the lack of relation with the test *Fluency*.

### LANGUAGE AND MATHEMATICS

The results of this study support the conclusion that vocabulary is related to mathematical performance (Alloway and Passolunghi, 2011). Our findings indicate that vocabulary makes a unique contribution to both *Arithmetic* and *Quantitative Concepts*, as these subtests have a high language load although these relations are strengthened when non-verbal intelligence is excluded from the regression analysis. As expected, language was not predictive of performance in the *Fluency* test given the low language level required by these operations. Therefore, our results suggest that vocabulary appears to be related to performance on those mathematical tests with high language requirements. The fact that language is related to mathematical performance must be taken into account for future research, challenging the traditional separation of these two key subjects in the schooling process. Most children who experience difficulties with mathematics also show language difficulties (e.g., Geary, 2011). This evident connection raises the question of whether a training program on language could improve concepts and word problem solving accuracy in typically developing children, an issue open to further research.

### SOCIOECONOMIC FACTORS AND MATHEMATICS

The current study contributes also to the existing literature on the relationship between parental educational levels and children’s performance in specific mathematical areas. The relationship of parental education and mathematical performance was observed only with the *Quantitative Concepts* test, and more specifically with the subtest *Concepts*. There are not enough studies currently to help us understand this apparently relevant relationship. Jordan and Levine (2009) have pointed out the importance of the socioeconomic and educational levels of parents on the mathematic achievement of their children, although that relationship was not studied in depth to determine whether it affects every aspect of mathematic performance. Aunio and Niemivirta (2010) related the educational level of parents to arithmetic abilities, suggesting that higher educational levels are predictors of better performance in children. Higher educational levels create an enriched environment in terms of language and literacy that supports the engagement of children in learning activities (e.g., Hart and Risley, 1995; Hoff-Ginsberg and Tardif, 1995; Kohl et al., 2000; Davis-Kean, 2005), which would account for the relationship between parental educational levels and *Quantitative Concepts* but not simple mathematical operations. This relationship is important, as studies such as that by Ohlsson and Rees (1991) state that poor understanding of the concepts underlying a procedure could be an obstacle to the development and acquisition of more complex procedures.

### LIMITATIONS AND FUTURE DIRECTIONS

The present findings suggest that different components of WM relate to different mathematical areas and that non-verbal intelligence and language may have different relationships depending on the mathematical area assessed. One limitation of the present study is that children who completed assessments in Times 1 and 2 had a lower IQ and their parents had a lower educational level than those who completed the assessment in Time 1 only. However, these missing data from children not tested in Time 2 were expected to increase the size of the observed associations between non-verbal intelligence and the rest of variables on one hand, and parental education and *Quantitative Concepts* on another hand, but not the observed pattern among them. Further research could help to determine whether the relationship among different WM components and specific mathematical areas is modulated by the kinds of tasks used in the present study and whether those relationships extend to a wider range of ages. Our results show that both language, and above all non-verbal intelligence, must also be taken into account in future studies exploring the relationship between WM and mathematics, given the association between language, non-verbal intelligence and mathematical performance.

### EDUCATIONAL IMPLICATIONS

The present findings have important implications regarding interventions for children with math learning difficulties, as they point out specific cognitive skills that could be trained in order to improve different aspects of mathematical ability. To the best of our knowledge, most training interventions such as Catch Up Numeracy or Numeracy Recovery (Dowker and Sigley, 2010; Holmes and Dowker, 2013) use similar mathematical exercises to those that serve to measure the different mathematical components children show some difficulties with, and therefore are mainly based on repetition and practice. This strategy may not be as useful for those children that their basic deficit is not on mathematics abilities *per se*, but on the lack of enough WM capacity to deal with and hold the necessary amount of partial information required for subsequent operations. We guess these children might benefit from training in specific WM components depending on what aspects of mathematics are to be improved, although one of the main problems with cognitive training is whether improvement leads to gains beyond the trained tasks (Mix and Cheng, 2012). Whether training in WM components would improve mathematical abilities in a direct way or through the improvement of fluid intelligence is a matter of further research. This latter pathway might be supported by the Jaeggi et al. (2008) study. The authors trained adults participants in a double (spatial and verbal) n-back task, a highly demanding task that taps the central executive of WM. Training not only improved performance in the n-back task but also generalized to fluid intelligence. Given the contribution that intelligence seems to have respect to the relationship between some WM components and mathematical performance, such training might have beneficial effects on mathematical abilities, but that is a question for further research.

## Conflict of Interest Statement

The authors declare that the research was conducted in the absence of any commercial or financial relationships that could be construed as a potential conflict of interest.

## References

[B1] Aiken L. S. West S. G. (1991). *Multiple Regression: Testing and Interpreting Interactions*. Newbury Park, CA: Sage

[B2] Alloway T. P. (2009). Working memory but not IQ predicts subsequent learning in children with learning difficulties. *Eur. J. Psychol. Assess.* 25 92–98 10.1027/1015-5759.25.2.92

[B3] Alloway T. P. Alloway R. G. (2010). Investigating the predictive roles of working memory and IQ in academic attainment. *J. Exp. Child Psychol.* 106 20–29 10.1016/j.jecp.2009.11.003 20018296

[B4] Alloway T. P. Gathercole S. E. Adams A. M. Willis C. S. (2005). Working memory abilities in children with special educational needs. *Educ. Child Psychol.* 22 56–67

[B5] Alloway T. P. Passolunghi M. C. (2011). The relations between working memory and arithmetical abilities: a comparison between Italian and British children. *Learn. Individ. Differ.* 21 133–137 10.1016/j.lindif.2010.09.013

[B6] Andersson U. (2008). Mathematical competencies in children with different types of learning difficulties. *J. Educ. Psychol.* 100 48–66 10.1037/0022-0663.100.1.48

[B7] Andersson U. Lyxell B. (2007). Working memory deficit in children with mathematical difficulties: a general or specific deficit? *J. Exp. Child Psychol.* 96 197–228 10.1016/j.jecp.2006.10.001 17118398

[B8] Aunio P. Niemivirta M. (2010). Predicting children’s mathematical performance in grade one by early numeracy. *Learn. Individ. Differ.* 20 427–435 10.1016/j.lindif.2010.06.003

[B9] Baddeley A. D. (1986). *Working Memory*. Oxford: Oxford University Press

[B10] Baddeley A. D. (2000). The episodic buffer: a new component of working memory? *Trends Cogn. Sci.* 4 417–423 10.1016/S1364-6613(00)01538-2 11058819

[B11] Berch D. B. (2008). Working memory and mathematical cognitive development: limitations of limited-capacity resource models. *Dev. Neuropsychol.* 33 427–446 10.1080/87565640801982494 18473207

[B12] Bull R. Johnston R. S. Roy J. A. (1999). Exploring the roles of the visual–spatial sketch pad and central executive in children’s arithmetical skills: views from cognition and developmental neuropsychology. *Dev. Neuropsychol.* 15 421–442 10.1080/87565649909540759

[B13] Castelli F. Glaser D. E. Butterworth B. (2006). Discrete and analogue quantity processing in the parietal lobe: a functional MRI study. *Proc. Natl. Acad. Sci. U.S.A.* 103 4693–4698 10.1073/pnas.0600444103 16537401 PMC1450233

[B14] Carlesimo G. A. Perri R. Turriziani P. Tomaiuolo F. Caltagirone C. (2001). Remembering what but not where: independence of spatial and visual working memory in the human brain. *Cortex* 37 519–534 10.1016/S0010-9452(08)70591-4 11721863

[B15] Cohen Kadosh R. Dowker A. Heine A. Kaufmann L. Kucian K. (2013). Interventions for improving numerical abilities: present and future. *Trends Neurosci. Educ.* 2 85–93 10.1016/j.tine.2013.04.001

[B16] Cornoldi C. Vecchi T. (2003). *Visuo-spatial Working Memory and Individual Differences*. New York, NY: Psychology Press

[B17] Davis-Kean P. (2005). The influence of parent education and family income on child achievement: the indirect role of parent expectations and the home environment. *J. Fam. Psychol.* 19 294–304 10.1037/0893-3200.19.2.294 15982107

[B18] Dehaene S. Spelke E. Pinel P. Stanescu R. Tsivkin S. (1999). Sources of mathematical thinking: behavioral and brain-imaging evidence. *Science* 284 970–974 10.1126/science.284.5416.970 10320379

[B19] Delazer M. (2003). “Neuropsychological findings on conceptual knowledge of arithmetic,” in *The Development of Arithmetic Concepts and Skill: Constructing Adaptive Expertise* eds Baroody A. J. Dowker A. (Hillsdale, NJ: Erlbaum) 385–408

[B20] Demeyere N. Rotshtein P. Humphreys G. W. (2012). The neuroanatomy of visual enumeration: differentiating necessary neural correlates for subitizing versus counting in a neuropsychological voxel-based morphometry study. *J. Cogn. Neurosci.* 24 948–964 10.1162/jocn_a_00188 22220729

[B21] De Smedt B. Taylor J. Archibald L. Ansari D. (2010). How is phonological processing related to individual differences in children’s arithmetic skills. *Dev. Sci.* 13 508–520 10.1111/j.1467-7687.2009.00897.x 20443971

[B22] Diamantopoulou S. Pina V. Valero-Garcia A. V. González-Salinas C. Fuentes L. J. (2012). Validation of the Spanish version of the Woodcock Johnson mathematics achievement tests for children aged six to thirteen. *J. Psychoeduc. Assess.* 30 466–477 10.1177/0734282912437531

[B23] Dowker A. (2001). Numeracy recovery: a pilot scheme for early intervention with young children with numeracy difficulties. *Support Learn.* 16 6–10 10.1111/1467-9604.00178

[B24] Dowker A. (2005). *Individual Differences in Arithmetic: Implications for Psychology, Neuroscience and Education*. Hove: Psychology Press 10.4324/9780203324899

[B25] Dowker A. Sigley G. (2010). Targeted interventions for children with arithmetical difficulties. *Br. J. Educ. Psychol. Monogr. Ser. II* 7 65–81 10.1348/97818543370009X12583699332492

[B26] Gaillard V. Barrouillet P. Jarrold C. Camos V. (2011). Developmental differences in working memory: where do they come from? *J. Exp. Child Psychol.* 110 469–479 10.1016/j.jecp.2011.05.004 21664622

[B27] Gathercole S. E. (1998). The development of memory. *J. Child Psychol. Psychiatry* 39 3–27 10.1017/S0021963097001753 9534084

[B28] Gathercole S. E. Adams A. M. Hitch G. J. (1994). Do young children rehearse? An individual differences analysis. *Mem. Cogn.* 22 201–207 10.3758/BF03208891 8035696

[B29] Geary D. C. (1993). Mathematical disabilities: cognitive, neuropsychological, and genetic components. *Psychol. Bull.* 114 345–362 10.1037/0033-2909.114.2.345 8416036

[B30] Geary D. C. (2011). Consequences, characteristics, and causes of poor mathematics achievement and mathematical learning disabilities. *J. Dev. Behav. Pediatr.* 32 250–263 10.1097/DBP.0b013e318209edef 21285895 PMC3131082

[B31] Gifford S. Rockliffe F. (2012). Mathematics difficulties: does one approach fit all? *Res. Math. Educ.* 14 1–15 10.1080/14794802.2012.657436

[B32] Grimm K. J. (2008). Longitudinal associations between reading and mathematics achievement. *Dev. Neuropsychol.* 33 410–426 10.1080/87565640801982486 18473206

[B33] Gross J. Hudson C. Price D. (2009). *The Long Term Costs of Numeracy Difficulties. Every Child a Chance Trust and KPMG*. East Sussex, UK: National Numeracy

[B34] Hale S. Bronik M. D. Fry A. F. (1997). Verbal and spatial working memory in school-age children: developmental differences in susceptibility to interference. *Dev. Psychol.* 33 364–371 10.1037/0012-1649.33.2.364 9147843

[B35] Hanley J. R. Young A. W. Pearson N. A. (1991). Impairment of the visuo-spatial sketch pad. *Q. J. Exp. Psychol.* 43 101–125 10.1080/14640749108401001 2017570

[B36] Hart B. Risley T. (1995). *Meaningful Differences in the Everyday Experiences of Young American Children*. Baltimore: Brookes

[B37] Hitch G. J. Halliday S. Schaafstal A. M Schraagen J. M. C. (1988). Visual working memory in young children. *Mem. Cogn.* 16 120–132 10.3758/BF03213479 3352517

[B38] Hoff-Ginsberg E. Tardif T. (1995). “Socioeconomic status and parenting,” in *Handbook of Parenting* Vol. 4 ed. Bornstein M. (Mahwah, NJ: Lawrence Erlbaum) 161–187

[B39] Holmes W. Dowker A. (2013). Catch up numeracy: a targeted intervention for children who are low-attaining in mathematics. *Res. Math. Educ.* 15 249–265 10.1080/14794802.2013.803779

[B40] Holmes J. Gathercole S. E. Dunning D. L. (2009). Adaptive training leads to sustained enhancement of poor working memory in children. *Dev. Sci.* 12 F9–F15 10.1111/j.1467-7687.2009.00848.x 19635074

[B41] Isaacs E. Vargha-Khadem F. (1989). Differential course of development of spatial and verbal memory span: a normative study. *Br. J. Dev. Psychol.* 7 377–380 10.1111/j.2044-835X.1989.tb00814.x

[B42] Jaeggi S. M. Buschkuehl M. Jonides J. Perrig W. J. (2008). Improving fluid intelligence with training on working memory. *Proc. Natl. Acad. Sci. U.S.A.* 105 6829–6833 10.1073/pnas.0801268105 18443283 PMC2383929

[B43] Jordan N. C. Levine S. C. (2009). Socioeconomic variation, number competence, and mathematics learning difficulties in young children. *Dev. Disabil. Res. Rev.* 15 60–68 10.1002/ddrr.46 19213011

[B44] Kaufman A. S. Kaufman N. L. (1990). *Kaufman Brief Intelligence Test* (Spanish translation by TEA Ediciones, Madrid). Bloomington, MN: Pearson

[B45] Kessels R. P. C. Postma A. Kappelle L. J De Haan E. H. F. (2000). Spatial memory impairments in patients after tumour resection: evidence for a double dissociation. *J. Neurol. Neurosurg. Psychiatry* 69 389–391 10.1136/jnnp.69.3.389 10945816 PMC1737096

[B46] Kessels R. P. C. Van den Berg E. Ruis C Brands A. M. A. (2008). The backward span of the Corsi block-tapping task and its association with the WAIS-III digit span. *Assessment* 15 426–434 10.1177/1073191108315611 18483192

[B47] Klingberg T. (2010). Training and plasticity of working memory. *Trends Cogn. Sci.* 14 317–324 10.1016/j.tics.2010.05.002 20630350

[B48] Kohl G. O. Lengua L. J. McMahon R. J. (2000). Parent involvement in school conceptualizing multiple dimensions and their relations with family and demographic risk factors. *J. School Psychol.* 38 501–523 10.1016/S0022-4405(00)00050-9 PMC2847291 20357900

[B49] Koponen T. Aunola K. Ahonen T. Nurmi J. E. (2007). Cognitive predictors of single-digit and procedural calculation skills and their covariance with reading skill. *J. Exp. Child Psychol.* 97 220–241 10.1016/j.jecp.2007.03.001 17560969

[B50] Krajewski K. Schneider W. (2009). Exploring the impact of phonological awareness, visual-spatial working memory, and preschool quantity-number competencies on mathematics achievement in elementary school: findings from a 3-year- longitudinal study. *J. Exp. Child Psychol.* 103 516–531 10.1016/j.jecp.2009.03.009 19427646

[B51] Lee K. Ng S. F. Ng E. L. Lim Z. Y. (2004). Working memory and literacy as predictors of performance on algebraic word problems. *J. Exp. Child Psychol.* 89 140–158 10.1016/j.jecp.2004.07.001 15388303

[B52] Levels M. Dronkers J. Kraaykamp G. (2008). Immigrant children’s educational achievement in western countries: origin, destination, and community effects on mathematical performance. *Am. Sociol. Rev.* 73 835–853 10.1177/000312240807300507

[B53] Logie R. H. (1995). *Visuospatial Working Memory*. Hove, UK: Lawrence Erlbaum

[B54] Luzzatti C. Vecchi T. Agazzi D. Cesa-Bianchi M. Vergani C. (1998). A neurological dissociation between preserved visual and impaired spatial processing in mental imagery. *Cortex* 34 461–469 10.1016/S0010-9452(08)70768-8 9669110

[B55] McLean J. F. Hitch G. J. (1999). Working memory impairments in children with specific arithmetic learning difficulties. *J. Exp. Child Psychol.* 74 240–260 10.1006/jecp.1999.2516 10527556

[B56] Mix K. S. Cheng Y. L. (2012). “The relation between space and math: developmental and educational implications,” in *Advances in Child Development and Behavior* Vol. 42 ed. Benson J. B. (Burlington, MA: Academic Press) 197–243 10.1016/b978-0-12-394388-0.00006-x 22675907

[B57] Muñoz-Sandoval A. F. Woodcock R. W. McGrew K. S. Mather N. (2005). *Bateria III Woodcock-Muñoz*. Rolling Meadows, IL: Riverside

[B58] Ohlsson S. Rees E. (1991). The function of conceptual understanding in the learning of arithmetic procedures. *Cogn. Instruct.* 8 103–179 10.1207/s1532690xci0802_1

[B59] Passolunghi M. C. Cornoldi C. (2008). Working memory failures in children with arithmetical difficulties. *Child Neuropsychol.* 1 1–14 10.1080/09297040701566662 18608224

[B60] Passolunghi M. C. Mammarella I. C. (2010). Spatial and visual working memory ability in children with difficulties in arithmetic word problem solving. *Eur. J. Cogn. Psychol.* 22 944–963 10.1080/09541440903091127

[B61] Passolunghi M. C. Mammarella I. C. (2012). Selective spatial working memory impairment in children with arithmetic learning disabilities. *J. Learn. Disabil.* 45 341–350 10.1177/0022219411400746 21444930

[B62] Passolunghi M. C. Pazzaglia F. (2005). A comparison of updating processes in children good or poor in arithmetic word problem-solving. *Learn. Individ. Differ.* 15 257–269 10.1016/j.lindif.2005.03.001

[B63] Passolunghi M. C. Vercelloni B. Schadee H. (2007). The precursors of mathematics learning: working memory, phonological ability and numerical competence. *Cogn. Dev.* 22 165–184 10.1016/j.cogdev.2006.09.001

[B64] Raghubar K. Barnes M. A. Hecht S. (2010). Working memory and mathematics: a review of developmental, individual difference and cognitive approaches. *Learn. Individ. Differ.* 20 110–122 10.1016/j.lindif.2009.10.005

[B65] Simmons F. R. Singleton C. H. Horne J. K. (2008). Phonological awareness and visual-spatial sketchpad functioning predict early arithmetic attainment: evidence from a longitudinal study. *Eur. J. Cogn. Psychol.* 20 711–722 10.1080/09541440701614922

[B66] Swanson H. L. (2011). Working memory, attention, and mathematical problem solving: a longitudinal study of elementary school children. *J. Educ. Psychol.* 103 821–837 10.1037/a0025114

[B67] Swanson H. L. Beebe-Frankenberger M. (2004). The relationship between working memory and mathematical problem solving in children at risk and not at risk for serious math difficulties. *J. Educ. Psychol.* 96 471–491 10.1037/0022-0663.96.3.471

[B68] Tam H. Jarrold C. Baddeley A. D. Sabatos-DeVito M. (2010). The development of memory maintenance: children’s use of phonological rehearsal and attentional refreshment in working memory tasks. *J. Exp. Child Psychol.* 107 306–324 10.1016/j.jecp.2010.05.006 20576275

[B69] Vandierendonck A. Kemps E. Fastame M. C. Szmalec A. (2004). Working memory components of the Corsi blocks task. *Br. J. Psychol.* 95 57–79 10.1348/000712604322779460 15005868

[B70] Wechsler D. (2004). *Manual for the Wechsler Intelligence Scale for Children,* 4th Edn, Traduccion al español. (TEA Ediciones, Madrid, 2005). San Antonio, TX: Harcourt

[B71] Zamarian L. Ischebeck A. Delazer M. (2009). Neuroscience of learning arithmetic evidence from brain imaging studies. *Neurosci. Biobehav. Rev.* 33 909–925 10.1016/j.neubiorev.2009.03.005 19428500

